# Pulmonary Artery Pressure in Patients with Markedly Deviated Septum Candidate for Septorhinoplasty

**Published:** 2014-07

**Authors:** Seyed Esmail Hassanpour, Seyed Mehdi Moosavizadeh, Mohsen Fadaei Araghi, Bahram Eshraghi

**Affiliations:** 1 Department of Plastic and Reconstructive Surgery, Shahid Beheshti University of Medical Sciences, Tehran, Iran;; 2 Department of Cardiology, Shahid Beheshti University of Medical Sciences, Tehran, Iran

**Keywords:** Nasal septum, Deviation, Septorhinoplasty, Pulmonary artery pressure

## Abstract

**BACKGROUND:**

The nasal septal deviation (NSD) is one of the major causes of nasal obstruction. This condition increases upper airway resistance. In This study we evaluated the mean pulmonary artery pressure (MPAP) in patients with markedly deviated septum.

**METHODS:**

Sixty two patients with NSD (Age range: 26-45 years, 34 men and 28 women) were included in the study. Mean pulmonary artery pressure was measured in preoperative period by Doppler echocardiography with the assistance of an expert cardiologist.

**RESULTS:**

The mean preoperative MPAP value (22.5 mmHg in men and 20.03 mmHg in women) of the patients in this study was higher than normal population (20 mmHg). The MPAP of nine patients (14.5%) was greater than 25 mmHg. This value was significantly higher than values for normal population.

**CONCLUSION:**

Markedly deviated septum had improper effects in cardiovascular system due to increase in MPAP.

## INTRODUCTION

The upper airway has been poorly understood by clinicians and respiratory physiologists and historically had been viewed as an amorphous structure between the nares and/or teeth and the trachea.^1^ The correlation of chronic upper airway obstruction in infants and children with pulmonary hypertension and right ventricular dysfunction has been well established and was first reported in the 1960.^2^

The nasal septum has a definitive impact on the function of the upper airway and nose. Seventy five to 80% of the general population is estimated to have some types of nasal deformity.^3^ The nose normally accounts for approximately half of the respiratory resistance to airflow.^3^ Nasal septal deviation is a major cause of nasal obstruction. Markedly deviated septum causes nasal obstruction that has been shown to decrease oxygen saturation and increase the arterial carbon dioxide content.^4^ The response to hypercarbia and acidosis is pulmonary vasoconstriction induced pulmonary hypertension.^5^ In the literature, the effect of chronic upper respiratory obstruction such as hypertrophied tonsils, adenoid vegetation and nasal polyposis on cardiopulmonary system has been studied but, the effect of pure nasal deviation on cardiac system has not precisely investigated. We aimed to assess the mean pulmonary artery pressure in patients with deviated septum candidate for septorhinoplasty.

## MATERIALS AND METHODS

Between July 2012 and July 2013, 62 patients with severe septal deviation who referred to Plastic Surgery Clinic of 15^th^ Khordad Hospital affiliated to Shahid Beheshti University of Medical Sciences in Tehran, Iran were enrolled. Para-nasal sinus tomography scan was performed for all patients. There was no other airway obstruction in patients.

Thirty four patients (54.8%) in the study group were male and 28 (45.1%) were female. A written informed consent was provided from all patients. Routine preoperative blood analysis was conducted for all patients. Past medical history of allergic rhinitis was obtained from the patients too.

Color Doppler echocardiography was used to estimate mean pulmonary artery pressure (Megas with Multifrequnce probe). We used a definite formula to estimate the mean pulmonary artery pressure [MPAP = 79 - 45/100 × Acceleration time (At)]. Acceleration time was the time necessary for pulmonary artery speed reach to peak. When the At was less than 120 milliseconds, we used another formula (MPAP = 90 - 62/100 × At).^6^^-^^8^ All patients underwent open septorhinoplasty under general anesthesia.

## RESULTS

Sixty two patients enrolled in this study (28 females and 34 males had 24-48 hours postoperative nasal tampon. Nine patients had obstructed nose in one side (unilateral breathing by nose). Seven of them were male and two of them were female. Fifteen patients had history of allergy and rhinorhea.

MPAP was greater than 25 mmHg for 9 patients (14.5%) and it was greater than 20 mmHg for 34 patients (54.8%). It was 20.03 mmHg in females, 22.5 mmHg for male patients. Considering all of the patients, it was 21.38 mmHg (Table 1).

**Table 1:** Characteristics of patients with markedly deviated septum

**Sex**	**Number**	**Mean age**	**Mean ** **pulmonary artery pressure**	**Obstructed nose**	**Allergy**
Male	34 (54.8%)	25.41	22.5	7 (11.3%)	4 (6.4%)
Female	28 (45.2%)	27.5	20.03	2 (3.2%)	11 (17.7%)

## DISCUSSION

Nasal septal deviation is frequently caused due to trauma or as a cause of congenital disorders. The nasal septum is the bone and cartilage of the nose that separates the nasal cavity into the two passages. Normally the septum lies centrally and thus the nasal passages are symmetrical. A deviated septum is an abnormal condition that can result in poor drainage of the sinuses. Patients may also have complain of difficulty in breathing headache, bloody nose and sleep disorder like snoring and sleep apnea. 

Nasal obstruction is known to disrupt the physiological ventilation of the lung by obstructing the airflow. When the air is not inhaled via nasal route, it is inhaled via oral route which increases the resistance of upper respiratory tract. It results in upper respiratory tract syndrome.^9^

Upper airway obstruction like nasal obstruction and deviation can cause complications due to hypoventilation. Many authors studied the development of cardiovascular complications in pulmonary arterial hypertension.^10^ Literature revealed report of increased pulmonary artery pressure after hypoxia associated pulmonary vasoconstriction resulting in ventricular hypertrophy due to overburden of the right cardiac ventricle which then led to right cardiac failure.^9^^,^^11^

Doppler echocardiography is a cost effective and non-invasive method being used to evaluate patients with pulmonary hypertension. Nevertheless, the majority of studies are concerned on chronic tonsillitis and adenoid vegetation cases^12^ but, the effect of nasal deviation on respiratory and cardiopulmonary system has not been investigated in detail. Some authors investigated this effect on cardiopulmonary system recently but, it is not complete.^13^ The upper limit of normal value of mean pulmonary artery pressure is 20 mmHg and we used Doppler echocardiography method in our study. In comparison to normal value, all data were in upper limit of normal value or increased. 

In this study, 34 out of 62 patients (54.8%) had a mean pulmonary artery pressure more than 20 mmHg and mean pulmonary artery pressure of both female and male groups were also greater than 20 mmHg. Based on our literature search, there were no reports on pulmonary hypertension associated with nasal deviation or obstruction, but the elevation of mean pressure more than normal in this study indicated that these patients impended to get pulmonary hypertension in the following years of their life. 

In conclusion, evaluation of cardio pulmonary system in patients with severe deviated septum or obstructed nose is very important. These patients should be encouraged to do septoplasty sooner for prevention of cardio-pulmonary complications.

## CONFLICT OF INTEREST

The authors declare no conflict of interest. 

## Footenote

Please cite this paper as:

Hassanpour SE, Moosavizadeh SM, Fadaei Araghi M, Eshraghi B. Pulmonary Artery Pressure in Patients with Markedly Deviated Septum Candidate for Septorhinoplasty. World J Plast Surg 2014;3(2):119-121.
